# High-Dose Nitrate Supplementation Attenuates the Increased Blood Pressure Responses to Isometric Blood Flow Restriction Exercise in Healthy Males

**DOI:** 10.3390/nu14173645

**Published:** 2022-09-03

**Authors:** Ozcan Esen, Ladislav Cepicka, Tomasz Gabrys, Raci Karayigit

**Affiliations:** 1 Department of Sport, Exercise and Rehabilitation, Northumbria University, Newcastle-upon-Tyne NE1 8ST, UK; 2 Department of Physical Education and Sport, Faculty of Education, University of West Bohemia, 30100 Pilsen, Czech Republic; 3 Department of Coaching Education, Faculty of Sport Sciences, Ankara University, Ankara 06830, Turkey

**Keywords:** nitric oxide, functional food, cardiovascular health, supplements, nutrition

## Abstract

The effect of nitrate (NO_3_^−^) supplementation on blood pressure (BP) responses during large muscle mass isometric and ischaemic exercise in healthy young adults is unclear. The aim of the present study was to assess the effect of 5-day supplementation of NO_3_^−^ on BP responses during a short isometric contraction and a sustained ischaemic contraction. In a randomised, double-blinded, crossover design, 14 healthy active young adults underwent BP measurements after 5 days of either NO_3_^−^ (NIT) or placebo (PLA) supplementation. Beat-by-beat BP was measured at pre- and post-exercise rest, and during a short (20 s) isometric contraction at 25% maximal strength and throughout a sustained ischaemic contraction. Plasma nitrite (NO_2_^−^) concentration increased significantly after NO_3_^−^ supplementation compared to placebo (475 ± 93 nmol·L^−1^ vs. 198 ± 46 nmol·L^−1^, *p* < 0.001, *d* = 3.37). Systolic BP was significantly lower at pre- (*p* = 0.051) and post-exercise rest (*p* = 0.006), during a short isometric contraction (*p* = 0.030), and throughout a sustained ischaemic contraction (*p* = 0.040) after NO_3_^−^ supplementation. Mean arterial pressure was significantly lower at pre- (*p* = 0.004) and post-exercise rest (*p* = 0.043), during a short isometric contraction (*p* = 0.041), and throughout a sustained ischaemic contraction (*p* = 0.021) after NO_3_^−^ supplementation. Diastolic BP was lower at pre-exercise rest (*p* = 0.032), but not at post-exercise rest, during a short isometric contraction, and during a sustained ischaemic contraction (all *p* > 0.05). Five days of NO_3_^−^ supplementation elevated plasma NO_2_^−^ concentration and reduced BP during a short isometric contraction and a sustained ischaemic contraction in healthy adults. These observations indicate that multiple-day nitrate supplementation can decrease BP at rest and attenuate the increased BP response during isometric exercise. These findings support that NO_3_^−^ supplementation is an effective nutritional intervention in reducing SBP and MAP in healthy young males during submaximal exercise.

## 1. Introduction

Beetroot juice supplementation has been used as a popular nutritional intervention, as its nitrate (NO_3_^−^)-rich content has documented ergogenic [1,2,3,4,5] and cardioprotective [6,7,8] effects. These effects of NO_3_^−^ supplementation have been attributed to its capacity to increase the bioavailability of nitric oxide (NO). NO is a gaseous signalling molecule that serves as an essential regulator in numerous physiological functions, such as preservation of metabolic and cardiovascular integrity [9]. NO is mainly produced through the oxidation of the amino acid L-arginine (via NO synthase (NOS) enzyme), with NO_3_^−^ and nitrite (NO_2_^−^) being inert end products of endogenous NO generation [10,11,12]. It is now known that NO can be also produced through the sequential reduction of NO_3_^−^ to NO_2_^−^, through anaerobic bacteria that reside in the oral cavity, and subsequently to NO [13].

The most well-documented influence of NO via supplementation of dietary NO_3_^−^ is to improve brachial artery flow-mediated dilation via activating soluble guanylate cyclase in the vascular smooth muscle [6,14,15,16], which regulates blood pressure (BP) [17]. Indeed, several studies reported that decreased BP following NO_3_^−^ supplementation is closely associated with increased plasma NO_2_^−^ levels in healthy [18,19] and trained populations [16,20]. The BP-lowering effect of NO_3_^−^ supplementation was well-reported during aerobic exercise in healthy and pre-hypertensives [21,22], and a few studies reported reduced BP responses during isometric arm exercise following acute NO_3_^−^ supplementation in hypertensives [23] and young adults [24]. Given NO has been shown to reduce sympathetic vasoconstriction in exercising skeletal muscle (sympatholysis) [25,26,27,28], it might be anticipated that increased NO bioavailability via NO_3_^−^ supplementation could lower sympathetic vasoconstriction during muscle contraction and alter sympathetic control of BP. Further, it has been reported that acute NO_3_^−^ supplementation reduced muscle sympathetic nerve activity during static handgrip exercise in young adults [24]. While the impact of NO_3_^−^ supplementation has been investigated, commonly with an acute regimen, on BP responses during muscle contraction, it is less clear how multiple-day NO_3_^−^ supplementation may affect BP responses to exercising muscle. Evaluating the effect of NO_3_^−^ supplementation, with a multiple-day regimen, on BP is important to improve understanding of the potential effect of NO_3_^−^ supplementations to modulate cardiovascular function during exercise. In addition, the effect of NO_3_^−^ supplementation on BP responses was mainly assessed during arm muscle isometric exercise, but its effect during large muscle mass (e.g., vastus lateralis) isometric exercise has not been investigated. Thus, further research is warranted to examine the influence of NO_3_^−^ supplementation on BP responses during large muscle mass contractions in humans.

Therefore, the aim of the present study was to assess the effect of 5-day supplementation of NO_3_^−^ on BP responses at rest before and after exercise and during leg muscle isometric exercise in healthy humans. In an attempt to maximise the conditions for conversion of NO_2_^−^ to NO [29,30], a sustained muscle contraction was completed with blood flow restriction which would exaggerate low PO_2_ and pH during contractions. It was hypothesised that 5-day NO_3_^−^ supplementation would: (1) increase plasma NO_2_^−^, (2) lower BP at rest pre- and post-exercise, and (3) attenuate the increased BP during isometric muscle contraction.

## 2. Materials and Methods

### 2.1. Participants

The sample size of this study was based on a prior calculation using G*Power software (version 3.1.9.4, Universität, Düsseldorf, Germany). A two-sided significance level of 0.05 and a power of 0.80 indicated that 10 participants would be sufficient to detect a difference in BP response, based on a small standardised effect size of 0.2 and the variance of the difference between PLA and NIT trials previously reported [31].

Fourteen males (mean ± SD age: 25 ± 6 years, stature: 174 ± 1 cm, mass: 78 ± 8 kg) volunteered in this study by providing written informed consent. Participants were injury-free and non-smokers. All participants self-reported that they were involved in regular moderate-intensity exercise ~3 days per week and muscle-strengthening activities ~2 days per week. The study received institutional ethical approval from the Manchester Metropolitan University Research Ethics Committee (reference no: 5951) and was conducted according to all aspects of the Declaration of Helsinki. Participants were requested to record their dietary intake five days prior to the first trial (throughout the first 5-day supplementation period) and to repeat the same diet five days prior to the second trial (throughout the second 5-day supplementation period). Individuals were also asked to maintain habitual physical activity and diet, refrain from antibacterial mouthwash, and abstain from caffeine, nutritional supplements, alcohol, and vigorous exercise (for 24 h) before trials.

### 2.2. Study Design 

In a randomised, double-blinded, crossover design, participants attended the laboratory at a similar time of day (±2) on two separate occasions to complete two experimental trials, one with 5-day supplementation of NO_3_^−^-rich beetroot juice (BRJ), and one with 5-day supplementation of NO_3_^−^-depleted BRJ. A washout period of 7 ± 1 days separated the supplementation periods. Randomisation was applied in counter-balanced fashion, in which the participant sample was divided in half, with one half completing the two conditions in one order and the other half completing the conditions in the reverse order. Randomisation and blinding were administered by an independent technician who did not take part in the assessments. Participants attended the testing facility after each 5-day supplementation period. In each visit, first a blood sample was collected, and resting BP was measured for 5 min. Then, maximum voluntary contractions (MVCs) of the knee extensors were performed by using a custom-built dynamometer [32] to determine individual 25% MVC for submaximal exercise protocol. The exercise protocol has been described elsewhere in detailed [33]. Briefly, a 20 s contraction at 25% MVC was performed firstly; then, a blood flow occlusion (BFO) period was applied at the proximal thigh (with 220 mmHg) for 8 min, with a sustained submaximal contraction at 25% MVC in the final 3 min of the BFO period. Restriction was then released, and the participant remained sitting for a further rest of 5 min.

### 2.3. Supplementation Procedures 

During the two 5-day supplementation periods, participants ingested 2 × 70 mL/day of concentrated NO_3_^−^-rich (NIT: ~12.8 mmol/day NO_3_^−^) or NO_3_^−^-depleted BRJ as placebo (PLA: ~0.08 mmol/day NO_3_^−^) (Beet It, James White Drinks Ltd., Ipswich, UK). Participants were instructed to supplement with the 2 × 70 mL shots around the same times each day (one in the morning at ~9 a.m. and one in the evening at ~9 p.m.) for the first 4 days of supplementation. On day five of supplementation, participants were instructed to ingest 2 × 70 mL shots together 2.5 h before the trial [33]. This regimen for multiple-day supplementation of NO_3_^−^ has been used as a standard approach in numerous previous studies (e.g., [1,2,20,33,34,35,36]). Participants were instructed to set up a time reminder for each time of the beetroot juice shots during a day and asked to keep a record of any days when they missed taking the beetroot juice, in order to monitor supplement compliance. Participants were also sent emails twice a day as reminders for beetroot juice ingestion. Given that the current recommendation for NO_3_^−^ supplementation is ~6–29 mmol/day of NO_3_^−^ [33,36,37], and the potential ergogenic effect of NO_3_^−^ supplementation by elevating plasma NO_2_^−^ concentration has been reported at the highest following dose of ~12.8 mmol/day in healthy humans [38], ~12.8 mmol/day of NO_3_^−^ was used in the present study.

### 2.4. Measurement

Beat-by-beat arterial BP: Following 10 min of rest of comfortable upright sitting, beat-by-beat BP was recorded by using finger photoplethysmography (Finometer, Finapres Medical Systems, Amsterdam, The Netherlands) [39]. BP was measured throughout the testing protocol (at pre-exercise rest, during short isometric contraction and the 3 min of sustained contraction with BFO, and at post-exercise rest). Systolic BP (SBP), mean arterial pressure (MAP), and diastolic BP (DBP) values were calculated as a mean of each phase using LabChart8 software (v8.1.13, Adintstruments Products, Oxford, UK).

Plasma NO_2_^−^: Venous blood samples were collected, at least 2.5 h after the last meal, to determine plasma NO_2_^−^ concentration. A venous blood sample was drawn into a lithium heparin tube (5 mL, Vacutainer, Becton Dickinson) at rest on both trial days. Samples were then centrifuged at 4000 rpm at 4 °C for 10 min (hettich^®^ 320 centrifuge, Tuttlingen, Germany). Plasma was subsequently extracted and immediately frozen at −80 °C for later analysis of NO_2_^−^ using a modification of the chemiluminescence technique as described elsewhere [16].

### 2.5. Statistical Analysis 

Paired *t*-tests were employed to test for differences between the NIT and PLA supplements in plasma NO_2_^−^ concentration, and BP (SBP, DBP, and MAPs) at pre- and post-exercise rest, and during short isometric contraction. The effect of NO_3_^−^ supplementation on the response of SBP, DBP, and MAP during the sustained ischaemic contraction was assessed by two-way repeated-measures ANOVAs. Effect sizes were calculated as partial eta squared (ŋ_p_^2^), varying from small (≥0.01), to moderate (≥0.06), and to large effect (≥0.14) [40]. When ANOVA revealed significant main effects and/or interaction effects, Bonferroni corrected paired *t*-tests were used as post-hoc paired comparisons. Cohen’s d effect sizes were determined for each paired comparison [40]. All data were analysed using SPSS 27.0 (IBM Corp., Armonk, NY, USA), and presented as mean ± SD. *p* value < 0.05 was considered statistically significant. Intraclass correlation coefficients (ICCs) were displayed with r values, and coefficient of variation (CV) is reported using standard deviation (SD)/mean × 100.

## 3. Results

Values for ICCs throughout the protocol (pre-exercise rest, brief contraction, sustained contraction, post-exercise) indicate a moderate reliability for SBP in NIT (ICC: *r* = 0.66, 95%, CV = 15.3%), DBS (ICC: *r* = 0.73, CV = 17.1%), and MAP (ICC: *r* = 0.70, CV = 20.9%); and a poor reliability for SBP (ICC: *r* = 0.48, CV = 14.2%), DBS (ICC: *r* = 0.52, CV = 15.9%) and MAP (ICC: *r* = 0.42, CV = 18.7%) in PLA.

**Figure 1:**
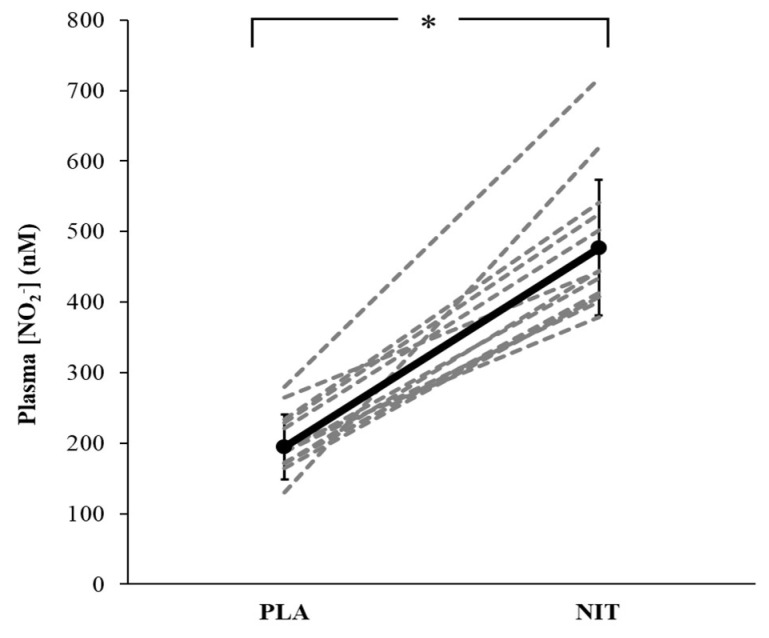
Group mean (SD) and individual plasma nitrite (NO_2_^−^) concentration responses after 5-day dietary nitrate (NIT) or placebo (PLA) supplementation are shown in the black and dashed lines, respectively. * *p*  <  0.05.

**Figure 2:**
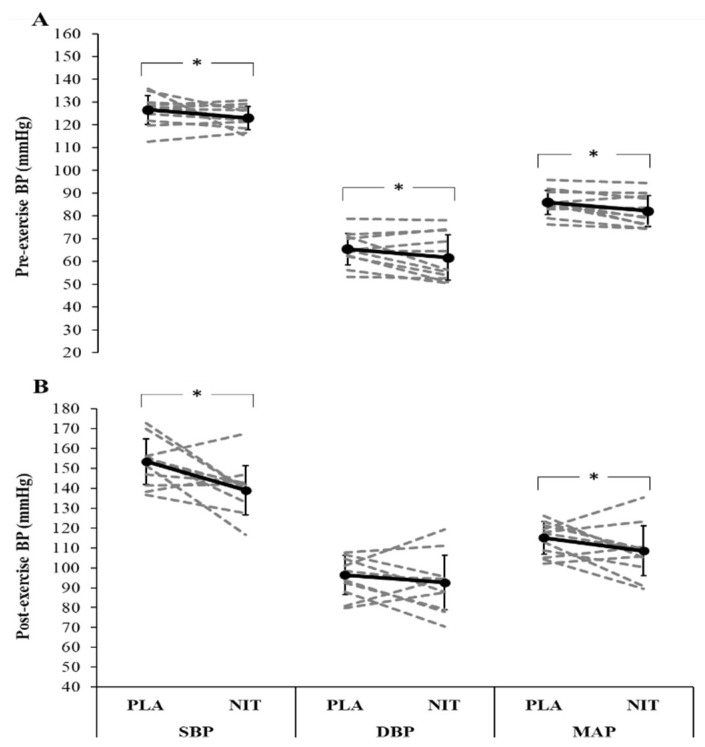
Blood pressure at pre-exercise rest (**A**), and post-exercise rest (**B**) in NIT and PLA trials. SBP: systolic blood pressure, DBP: diastolic blood pressure, MAP: mean arterial pressure. Data are mean ± SD. * *p* < 0.05.

**Figure 3:**
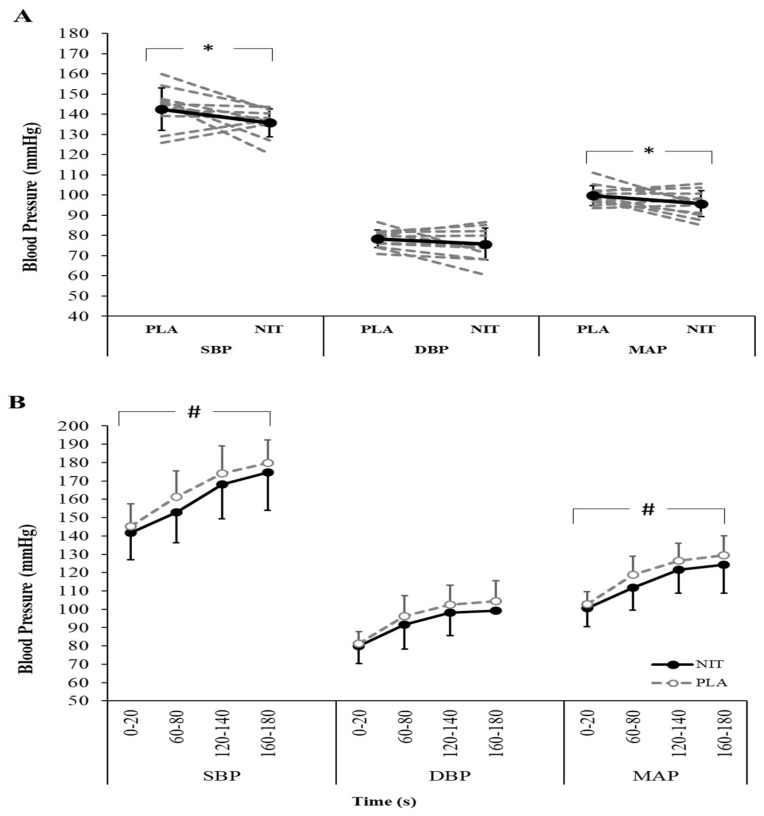
Systolic blood pressure (SBP), diastolic blood pressure (DBS), mean arterial pressure (MAP) during short isometric contraction (**A**) and a sustained ischaemic contraction (**B**) following nitrate (NIT) and placebo (PLA) supplementation. Data are mean ± SD. * *p* = 0.030 for SBP and *p* = 0.041 for MAP. # Main effect of supplementation, *p* = 0.040 for SBP and *p* = 0.021 for MAP.

NIT resulted in significantly higher plasma NO_2_^−^ concentration compared with the PLA trial (477 ± 95 nmol·L^−1^ vs. 195 ± 46 nmol·L^−1^, *p* < 0.001, *d* = 3.78, 95% CI [1.86, 4.50], Figure 1). The group mean SBP, DBP, and MAP responses in pre- and post-exercise rests, and during short contraction and sustained ischaemic contraction (at 0–20, 60–80, 120–140, and 160–180 s) following both NO_3_^−^ and placebo supplementation are presented in Figure 2 and Figure 3, respectively. The mean values for SBP, DBP and MAP were also reported in Supplementary Table S1. Compared to PLA, NIT resulted in a significant reduction in SBP (*p* = 0.051, *d* = 0.72, 95% CI [−0.09, 1.20]), DBP, (*p* = 0.032, *d* = 0.6, 95% CI [−0.05, 1.14]), and MAP (*p* = 0.004, *d* = 0.66, 95% CI [−1.57, −0.22]) at pre-exercise rest (Figure 2A). SBP (*p* = 0.006, *d =* 1.27, 95% CI [0.17, 1.50]) and MAP (*p* = 0.043, *d =* 0.56, 95% CI [−0.08, 1.14]) were significantly lower at post-exercise rest in the NIT compared to the PLA trial, whereas DBP did not differ significantly between trials (*p* = 0.150, *d =* 0.25, 95% CI [−0.07, 1.10], Figure 2B).

SBP (*p* = 0.030, *d* = 0.45, 95% CI [−0.04, 1.20]) and MAP (*p* = 0.041, *d =* 54, 95% CI [−0.07, 1.15]) were also significantly lower after the NIT trial compared to the PLA trial, whereas DBP did not differ between trials during short isometric contraction (*p* = 0.101, *d* = 0.31, 95% CI [−0.21, 0.94], Figure 3A).

SPB increased during the 3 min sustained ischaemic contraction (ANOVA: time, F = 61.54, *p* < 0.01, ŋ_p_^2^ = 0.848) and this increase was significantly lower in the NIT trial compared to the PLA trial (ANOVA: supplementation, F = 5.44, *p* = 0.040, ŋ_p_^2^ = 0.331), without supplementation × time interaction effect (F = 0.54, *p* = 0.617, ŋ_p_^2^ = 0.047). MAP increased during the 3-min sustained ischaemic contraction (ANOVA: time, F = 78.84, *p* < 0.01, ŋ_p_^2^ = 0.878), and this increase was significantly lower in the NIT trial compared to the PLA trial (ANOVA: supplementation, F = 7.29, *p* = 0.021, ŋ_p_^2^ = 0.399), without supplementation × time interaction effect (F = 0.53, *p* = 0.578, ŋ_p_^2^ = 0.046). DBP increased during the 3 min sustained ischaemic contraction (ANOVA: time, F = 44.69, *p* < 0.01, ŋ_p_^2^ = 0.802), but this increase was not significantly different between the NIT and PLA trials (ANOVA: supplementation, F = 3.24, *p* = 0.10, ŋ_p_^2^ = 0.227; ANOVA: supplementation × time interaction, F = 0.45, *p* = 0.598, ŋ_p_^2^ = 0.039).

## 4. Discussion

The present study examined the influence of multiple-day supplementation of NO_3_^−^ on BP responses during short isometric knee extensor contraction and a 3 min sustained ischaemic contraction in healthy young males. The main findings of the present study were that 5 days of NO_3_^−^ supplementation (~12.8 mmol/d^−1^ of NO_3_^−^) (I) elevated plasma NO_2_^−^ concentration, (II) lowered BP at pre- and post-exercise rest, and (III) attenuated the rise in BP during short isometric contraction and a sustained ischaemic contraction in healthy adults. These findings have extended the knowledge regarding the BP-lowering impact of NO_3_^−^ with multiple-day supplementation during isometric and ischaemic exercise in healthy adults using a double-blind, placebo-controlled, crossover design.

Plasma NO_2_^−^ concentration was elevated following 5 days of NO_3_^−^ supplementation, and this elevation was 245% higher compared to placebo, which suggests considerably improved potential for NO bioavailability via reduction of NO_2_^−^ to NO [13,41]. This result is in line with previous reports (e.g., [1,2,18,19,20,33]) and indicates the potential for NO bioavailability to reduce exercise-induced augmentation of BP [18,19,23,42].

BP was lower in the NIT trial compared with the PLA trial at rest and during short isometric muscle contraction. The findings of the present study on BP at rest are consistent with previous reports in healthy active individuals [16,18,19,20]. However, the observed effect on BP during short muscle contraction is inconsistent with a previous study by de Vries et al. [43] that reported no effect of acute NO_3_^−^ supplementation on a contracting large muscle mass in young healthy adults. Since we administered the dose of ~12.8 mmol/day of NO_3_^−^ over multiple days (5 days), it is possible that the duration of supplementation may have contributed to the effects observed. Indeed, a greater effect of NO_3_^−^ supplementation has been reported following multiple-day compared to acute supplementation [34]. 

Although isometric knee extension exercise with BFO increased BP following both NO_3_^−^ and placebo supplementation, there was a markedly lower increase in BP following NO_3_^−^ supplementation. While this effect was observed during the short contraction, it became evident from the first 20 s of the sustained ischaemic contraction. These findings are consistent with previous observations reporting reduced BP during submaximal handgrip exercise after NO_3_^−^ supplementation in hypertensive patients [23] and during dynamic (aerobic) exercise in healthy individuals [21,22]. It is important to highlight several important differences in the present study compared to previous investigations. While we applied 5-day high-dose supplementation in healthy participants, the BP-reducing effect of NO_3_^−^ was reported in hypertensives after a single dose of dietary NO_3_^−^ ingestion [23,44]. Interestingly, larger reductions in BP after dietary NO_3_^−^ were reported in individuals with chronic disease than in healthy individuals [44], and these reductions were greater in individuals with higher resting BP values [45]. We also applied the isometric exercise to a large muscle mass (leg) while others used a small muscle mass [23,44,45]. Regarding muscle mass, Polito et al. [46] reported a greater reduction in SBP after 10 sets of 10 reps of leg extension exercise when compared to elbow flexion exercise in normotensive men, suggesting the amount of muscle mass activated during exercise has an impact on BP. Together, these findings suggest (I) that the supplementation regimen of nitrate needs to be considered and/or managed depending on an individual’s health status, and (II) that the exercising muscle mass/group might have an impact on the efficacy of NO_3_^−^ supplementation, at least for BP responses. As mentioned above, the effect of NO_3_^−^ supplementation on BP is mostly related to improved systematic NO bioavailability [18,19,23,42], and BFO likely leads to exaggerated hypoxic conditions during muscle contraction, which would be expected to elicit the reduction of NO_2_^−^ to NO [29,30]. Further, existing evidence shows that NO_3_^−^ supplementation may enhance post-ischaemic vasodilation in healthy young adults after both acute and chronic administration [15,47,48], suggesting improved endothelial function, which may explain lower BP response after NO_3_^−^ supplementation compared to placebo during post-exercise rest. Taken together, these findings might explain the positive influence of NO_3_^−^ supplementation on BP during the sustained contraction with BFO in the present study. The effects of NO_3_^−^ supplementation have been reported to be more apparent in less fit or recreationally active individuals [49], likely due to their lower baseline plasma NO_2_^−^ levels. Since the participants in the present study were all recreationally active and since elevated plasma NO_2−_ levels were observed after NO_3_^−^ supplementation, it is plausible that NO_3_^−^ supplementation increased NO bioavailability at the vascular level and thus induced the reducing effect on BP.

Lowered BP is attributed to improved vascular tone (vasodilation) via increased cGMP after NO_3_^−^ supplementation [14,15,16]. Elevated NO bioavailability via NO_3_^−^ supplementation appears to increase cGMP [16], which might accelerate the removal of calcium in the vascular smooth muscle, leading to vasodilation [50]. In addition, since there is some evidence that NO may inhibit sympathetic vasoconstriction in resting and contracting skeletal muscle (sympatholysis) [25,26,27,28], lowered BP at rest and during short muscle contraction following NO_3_^−^ supplementation might be linked to reduced sympathetic vasoconstriction and altered sympathetic control of BP. Given that the sympathetic nervous system can also influence pressure and endothelium-dependent vasodilation [51,52], reduced BP via increased NO bioavailability might be also linked to alterations in efferent sympathetic outflow [53,54]. This view is supported by studies reporting that NO_3_^−^ supplementation has (I) lowered muscle sympathetic nerve activity during a 2 min isometric handgrip exercise [25], and (II) reduced sympathetic signalling during aerobic exercise [21]. 

## 5. Limitations

Several important points must be considered when interpreting the findings of this study. The current investigation did not include females. However, since different plasma NO_3_^−^ and BP responses between males and females have been reported [19], the findings of the present study can only be interpreted for males, and future studies should include females to investigate if the potential effect of NO_3_^−^ supplementation in altering BP is different compared with males, and to what extent. Though it remains to be elucidated fully, there are multiple proposed mechanisms for the BP-lowering effect of NO_3_^−^ supplementation; however, we did not measure any mechanism of action-related parameters (e.g., blood flow and/or sympathetic nerve activity) in this investigation. Future studies should measure these parameters independently or in combination to improve understanding of the mechanisms of the BP-lowering effect of NO_3_^−^ supplementation during exercise. It is also important to note that acute effects due to doubling the dose (2 × 70 mL shots together) on the testing day cannot be ruled out. Therefore, future protocols should consider a longer-term intervention (longer than 8 weeks), without the need to further supplement on the testing day, as the longer duration would have already allowed the supposed BP and associated circulatory adaptations. We could not control participants’ dietary intake but relied instead on the participants recording their dietary intake for 5 days before (throughout the first 5-day supplementation period) the first trial and replicating this before the subsequent trial (throughout the second 5-day supplementation period). While the participants reported that they had complied with this requirement, since it was self-reported, monitoring of dietary intake can be considered a limitation. Thus, future studies might control pre-test diet more rigorously. Since it was self-reported, monitoring of supplement compliance can be also considered a limitation, although the rate of supplement compliance was 100%. There is plenty of evidence to suggest that nitrate can improve exercise and/or physical capacity, which can facilitate the regulation of BP itself [33,35,36]. Given that this study focused on BP responses during exercise, but not exercise or/and physical parameters, we cannot exclude the possibility that some effects could be due to supplement-induced improved exercise and/or physical capacity instead of the direct effect of supplementation on BP. Nevertheless, the findings of this study provide novel data which will be of interest to those wanting to know the effects of NO_3_^−^ on blood pressure during exercise. 

## 6. Conclusions

The findings reveal that short-term high-dose NO_3_^−^ supplementation elevated plasma NO_2_^−^ concentration and reduced BP during a short isometric contraction and a sustained ischaemic contraction in healthy males. These findings support that NO_3_^−^ supplementation is an effective nutritional intervention in reducing SBP and MAP in healthy young males during submaximal exercise.

## Data Availability

Data are available for research purposes upon reasonable request to the corresponding author.

## Supplementary Materials

The following supporting information can be downloaded at: https://www.mdpi.com/article/10.3390/nu14173645/s1, Table S1: Systolic blood pressure (SBP), diastolic blood pressure (DBS), mean arterial pressure (MAP) responses at pre- and post-exercise rests, and during short isometric contraction and the sustained ischemic contraction.
