# Supplementary material for: High-Dose Nitrate Supplementation Attenuates the Increased Blood Pressure Responses to Isometric Blood Flow Restriction Exercise in Healthy Males
Source: Nutrients. 2022 Sep 3;14(17):3645. doi: 10.3390/nu14173645 (PMC9460709; doi:10.3390/nu14173645)
Supplement: Supplementary file 1 [file nutrients-14-03645-s001.zip › nutrients-1890728-supplementary.pdf]

Table S1. Systolic blood pressure (SBP), diastolic blood pressure (DBS), mean arterial pressure (MAP) responses at pre- and post-exercise rests, and during short isometric contraction and the sustained ischemic contraction. SBP: systolic blood pressure, DBP: diastolic blood pressure, MAP: mean arterial pressure, NIT: nitrate supplementation, PLA: placebo supplementation, *: $P < 0.05$, #: ANOVA: the main effect of supplementation.

BP (mmHg)	PLA	NIT
**Pre-exercise rest**		
SBP	127 ± 6	123 ± 5*
DBP	66 ± 7	62 ± 10*
MAP	86 ± 5	82 ± 7*
**Short contraction**		
SBP	142 ± 11	137 ± 7*
DBP	78 ± 4	76 ± 8
MAP	99 ± 5	96 ± 6*
**Ischemic contraction**		
**160-180 s**		
SBP	180 ± 13	175 ± 21#
DBP	105 ± 11	99 ± 17
MAP	130 ± 11	124 ± 16#
**120-140 s**		
SBP	174 ± 15	168 ± 19#
DBP	103 ± 11	98 ± 13
MAP	126 ± 9	122 ± 12#
**60-80 s**		
SBP	161 ± 14	153 ± 17#
DBP	96 ± 11	92 ± 13
MAP	118 ± 11	112 ± 12#
**0-20 s**		
SBP	145 ± 12	142 ± 15#
DBP	81 ± 7	80 ± 10
MAP	103 ± 7	101 ± 10#
**Post-exercise rest**		
SBP	153 ± 11	139 ± 11*
DBP	96 ± 10	93 ± 14
MAP	115 ± 8	109 ± 13*
